# Thyroid psychosis: a case report

**DOI:** 10.1192/j.eurpsy.2022.2035

**Published:** 2022-09-01

**Authors:** A. Izquierdo De La Puente, P. Del Sol Calderón, M. Garcia Moreno

**Affiliations:** 1 HOSPITAL UNIVERSITARIO PUERTA DE HIERRO MAJADAHONDA, Psychiatry, MADRID, Spain; 2 Hospital Universitario Puerta de Hierro, Psiquiatría Infanto-juvenil, Madrid, Spain

**Keywords:** Psychosis, Thyroid

## Abstract

**Introduction:**

We present the case of a patient who after a year of psychotic symptoms is diagnosed with thyroid cancer with hyperthyroidism.

**Objectives:**

A brief review is made of the psychotic symptoms in a patient with hyperthyroidism secondary to cancer of the gland.

**Methods:**

We present the case of a 52-year-old patient, a former injecting drug addict, who after a year with psychotic symptoms, is diagnosed with thyroid cancer with hyperthyroidism. The patient reported that a year ago, he suddenly had a painless and indurated lump in his neck, associated with weight loss and confusional symptoms. One month after the appearance of the tumor, the patient began to present visual, kinesthetic and haptic hallucinations, with the sensation that supernatural beings were passing through and possessing him. Likewise, he referred to being able to see and feel the atoms of matter, being able to communicate with a superior being whom he called “creator”.

**Results:**

The patient is admitted for psychotic symptoms. During it, the necessary complementary tests are carried out, objectifying a clinical situation of hyperthyroidism. The study is extended, observing a hyperfunctioning nodule, which corresponded to thyroid cancer.

**Conclusions:**

Neuropsychiatric symptoms in hyperthyroidism are relatively common. In most cases, the most frequent are cognitive alterations, attention problems and working memory problems. It can also lead to depressive episodes, and more rarely, psychotic symptoms.

**Disclosure:**

No significant relationships.

